# Advanced Imaging Techniques for Differentiating Pseudoprogression and Tumor Recurrence After Immunotherapy for Glioblastoma

**DOI:** 10.3389/fimmu.2021.790674

**Published:** 2021-11-25

**Authors:** Yan Li, Yiqi Ma, Zijun Wu, Ruoxi Xie, Fanxin Zeng, Huawei Cai, Su Lui, Bin Song, Lei Chen, Min Wu

**Affiliations:** ^1^ Huaxi MR Research Center (HMRRC), Department of Radiology, Functional and Molecular Imaging Key Laboratory of Sichuan Province, West China Hospital, Sichuan University, Chengdu, China; ^2^ Department of Clinic Medical Center, Dazhou Central Hospital, Dazhou, China; ^3^ Laboratory of Clinical Nuclear Medicine, Department of Nuclear Medicine, West China Hospital, Sichuan University, Chengdu, China; ^4^ Department of Neurology, West China Hospital, Sichuan University, Chengdu, China; ^5^ Department of Clinical Research Management, West China Hospital, Sichuan University, Chengdu, China

**Keywords:** glioblastoma, immunotherapy, treatment response, pseudoprogression, tumor recurrence, advanced imaging

## Abstract

Glioblastoma (GBM) is the most common malignant tumor of the central nervous system with poor prognosis. Although the field of immunotherapy in glioma is developing rapidly, glioblastoma is still prone to recurrence under strong immune intervention. The major challenges in the process of immunotherapy are evaluating the curative effect, accurately distinguishing between treatment-related reactions and tumor recurrence, and providing guidance for clinical decision-making. Since the conventional magnetic resonance imaging (MRI) is usually difficult to distinguish between pseudoprogression and the true tumor progression, many studies have used various advanced imaging techniques to evaluate treatment-related responses. Meanwhile, criteria for efficacy evaluation of immunotherapy are constantly updated and improved. A standard imaging scheme to evaluate immunotherapeutic response will benefit patients finally. This review mainly summarizes the application status and future trend of several advanced imaging techniques in evaluating the efficacy of GBM immunotherapy.

## Introduction

Glioblastoma is the most common malignant brain tumor in adult and is extremely aggressive. The current standard treatment involves maximal safe resection, followed by radiotherapy and adjuvant chemotherapy (1). Despite this active treatment, the prognosis remains poor, with a median survival of less than 2 years (2). The main reason is that glioblastoma is strongly aggressive and grows rapidly, specifically, tumor cells are prone to infiltrate the normal brain parenchyma aside the lesion (3, 4). Thus, there is a risk of tumor recurrence once tumor stem cells remain after the resection and follow-up treatment. Many other treatments have been studied, such as immunotherapy, aiming to stimulate or mobilize the immune system and enhance the antitumor immunity in the tumor microenvironment, so as to control and kill tumor cells (5). This treatment concept has derived a variety of treatment strategies, and remarkable progress of those methods has been made in the treatment of patients with intractable solid tumors such as melanoma and nonsmall cell lung cancer (6, 7). There are also many immunotherapy studies of glioma not only basic but also clinical. Due to the existence of blood-brain barrier (BBB) in the central nervous system (8), obvious loss of lymphatic reflux system (9), and the strong heterogeneity of GBM (10), the effectiveness of immunotherapy for brain tumors might be limited. Fortunately, it has been found that immunotherapy has the potential to induce immune changes in brain tumors (11, 12).

One of the challenges in the treatment of GBM is how to assess the treatment response accurately in order to make more informed clinical decisions. It is important to evaluate the treatment response to immunotherapy in an early stage by using noninvasive imaging, which can reduce unnecessary clinical complications. However, conventional imaging techniques are usually difficult to distinguish between pseudoprogression and tumor recurrence. Immune response is usually accompanied with inflammatory reaction characterized by the enlargement of enhanced foci, which is easily confused with the behavior of tumor relapse. Effective immunotherapy may be mistakenly terminated if being misdiagnosed, thus causing a negative impact on the prognosis. To solve this problem, researchers have carried out a lot of researches on advanced imaging techniques. This review describes the definition and clinical significance of pseudoprogression, generalizes the response evaluation criteria of GBM, summarizes the status and future development direction of advanced imaging techniques relevant to immunotherapy in GBM, and discusses the strengths and deficiencies of artificial intelligence (AI) in monitoring therapeutic response in GBM.

## Pseudoprogression of GBM

About 30% of GBM patients who received radiotherapy and adjuvant temozolomide-based chemotherapy had pseudoprogression, which mainly occurred within 3 months after treatment (13). According to the Response Assessment in Neuro-Oncology (RANO) criteria, pseudoprogression was defined as the appearance of new lesion or an increase in contrast-enhancing areas, but these changes gradually faded or stabilized without changing the treatment (14). At present, it is believed that the enlargement of enhanced foci may be caused by the infiltration of inflammatory factors after radiotherapy and chemotherapy, but the real cause of pseudoprogression remains to be further studied. In addition, the methylation status of the O6-methylguanine-DNA methyltransferase (MGMT) promoter was associated with pseudoprogression, and about 2/3 of GBM patients with MGMT methylation exhibited pseudoprogression (15).

Patients with pseudoprogression usually have no clinical symptoms and only show new or enlarged enhanced lesions on images. Such patients usually only need symptomatic treatment and do not need to change the treatment project, while patients with tumor recurrence probably need to resect the lesion again or find another cure. If there is no accurate distinction between them, the effectiveness of treatment may be reduced. Therefore, correct identification of pseudoprogression and tumor recurrence is of great significance to guide clinical decision-making.

## Response Evaluation Criteria of GBM

Noninvasive imaging for GBM can help define widely applicable treatment response criteria to assess disease progression and make clinical decisions. In order to address imaging challenges such as pseudoprogression, multidisciplinary experts developed RANO criteria (14), which suggested that the original treatment regimen can be maintained for patients with no clear clinical symptoms and only tumor progression on imaging. These patients only need regular follow-up. At present, the RANO criteria have been widely accepted in the field of neuro-oncology and applied in clinical and scientific researches. However, evaluating the therapeutic response to immunotherapy only by RANO criteria may not be sufficient. For example, the mechanism of pseudoprogression caused by immunotherapy may be different from that of standard therapy, which may be due to the infiltration of immune cells and inflammatory cells. It is necessary to establish corresponding imaging response criteria for immunotherapy in GBM.

Based on the important factors above, experts developed immunotherapy Response Assessment in Neuro-Oncology (iRANO) criteria for patients with GBM receiving immunotherapy to provide guidance for imaging changes in the early stage of progression (16). According to the iRANO criteria, the time window for pseudoprogression after immunotherapy is 6 months. Hence, the criteria recommend that patients with no significant clinical symptoms and evidence of early imaging progress within 6 months after immunotherapy should continue to receive immunotherapy before follow-up imaging confirms the tumor progression. In other words, patients with evidence of imaging progress outside the time window after immunotherapy will have a higher probability of potential true tumor progression, and these patients should be advised to discontinue ongoing immunotherapy.

## Application of Advanced Imaging in Immunotherapy of GBM

At present, the researches of glioma immunotherapy strategy mainly include the following: (1) specific peptide vaccine; (2) immunotoxin therapy; (3) immune checkpoint inhibitors (ICIs) therapy; (4) dendritic cell (DC) therapy; and (5) chimeric antigen receptor T-cell (CAR-T) Immunotherapy (17–21). The feasibility and safety of DC vaccine in the treatment of glioma have been proved, and it could induce immune response (20). It is worth noting that a new type of gamma delta T (γδ T)-cell therapy is becoming a rising star of cancer immunotherapy (22). Unlike the alpha beta T (αβ T) cells involved in most T-cell researches and clinical applications, γδ T cells recognize their target cells independently of major histocompatibility complex (MHC) and do not cause graft-*versus*-host disease. γδ T cells infiltrate in a variety of tissues, which can quickly respond to the target cells and release effector cytokines. Furthermore, the recognition and killing of tumor by γδ T cells do not depend on the expression of single antigen (23). Based on the advantages of γδ T cells, a new CAR-T therapy can be developed to break through the limited application of αβ T-cell-based CAR-T-cell therapy in solid tumors (including gliomas) (24, 25). Currently, γδ T-cell therapy has been studied in the treatment and prevention of recurrence of solid tumors including head and neck cancer, breast cancer, and lung cancer (26–28). The therapeutic effect in glioma still needs to be verified in a large number of clinical trials.

Advanced imaging techniques based on physiological or metabolic characteristics may reflect the state of tumor more accurately, so various advanced imaging techniques are being studied to correctly identify immunotherapy-related changes and tumor progression and provide a credible basis for the treatment of patients. The advanced imaging techniques used in GBM currently include perfusion-weighted imaging (PWI), diffusion imaging, amide proton transfer (APT), magnetic resonance spectroscopy (MRS), positron emission tomography (PET) (i.e., **Table 1**). Some of these imaging techniques have been used to evaluate the immunotherapy efficacy of glioma. The following will introduce the basic concepts of these imaging techniques and describe the latest research progress and future application prospects that support them in the evaluation of therapeutic response to immunotherapy.

**Table 1:** Studies of applying advanced imaging techniques to assess immunotherapeutic responses in GBM.

References	Advanced imaging	Evaluation parameters	Tumor type	Immunotherapy category	Evaluation criteria
(29)	DSC-MRI	ΔrCBVmax	GBM	DC vaccination	RANO
	DWI-MRI	rADC			
(30)	DSC-MRI	Maximum lesional rCBV ratios	Recurrent GBM	DC vaccination	Macdonald
	DWI-MRI	Minimum ADC			
(31)	DSC-MRI	rCBV	GBM	Immunogene-treated	NA
(32)	DCE-MRI	Ve	GBM (rats)	mAb9.2.27+NK	NA
(33)	DSC-MRI	Interval change in rADC	Recurrent GBM	ICIs	mRANO
	DWI-MRI				
(34)	DWI-MRI	Serial parametric response mapping of ADC	Pediatric diffuse intrinsic pontine glioma	Peptide-based vaccine	NA
(35)	DWI-MRI	IADC VOI	Recurrent GBM	ICIs	RANO
					Pathological
(36)	DWI-MRI	RSI	GBM	ICIs	Pathological
(37)	MRS	Cho, NAA, Crea, Lac	GBM	IL-4 toxin	Pathological
(38)	Amino acid PET	18F-FET PET/CT	GBM	DC vaccination	RANO
(39)	dck PET	[18F]-CFA PET/CT	GBM (human)	DC vaccination and/or PD-1 mAb blockade	NA
		[18F]-FAC PET/CT	Orthotopic malignant gliomas (mice)		

Ve, extravascular extracellular space volume fraction; IADC, intermediate ADC; VOI, volumes of interest; mAb9.2.27, a monoclonal antibody-targeting NG2; NK, natural killer cells; IL-4, interleukin 4; mRANO, modified RANO.

### Perfusion-Weighted Imaging

PWI can reflect tissue perfusion by quantitatively calculating perfusion parameters including relative cerebral blood volume (rCBV), relative cerebral blood flow (rCBF), mean transit time (MTT), and time to peak (TTP). When the tumor progresses, neovascularization and increased perfusion could be observed in the lesion area. As pseudoprogression is usually caused by inflammation, there is no neovascularization and the perfusion is relatively low. As a consequence, these perfusion parameters can be used to distinguish between pseudoprogression and tumor recurrence in GBM patients receiving standard treatment or immunotherapy (30, 40).

DSC-MRI is the most commonly used perfusion technique in clinic. Evidence has shown that adding perfusion imaging to conventional MRI in patients with gliomas is helpful for clinical decision-making (41, 42). A recent meta-analysis including 35 studies on the role of various advanced imaging techniques in evaluating the therapeutic response of high-grade gliomas indicated that the diagnostic accuracy of perfusion imaging was only second to MR spectroscopy (MRS). The sensitivity and specificity of DSC were 87% and 86%, respectively, while the sensitivity and specificity of DCE were 92% and 85%, respectively (43). In addition, a retrospective study comparing the value of DSC-MRI and DCE-MRI combined with T1WI enhancement and DWI imaging in predicting the recurrence of GBM revealed that both the two perfusion imaging could significantly improve the diagnostic accuracy, and there was no significant difference in diagnostic performance (42). Similarly, some studies have compared the diagnostic accuracy of DSC-MRI with three-dimensional pseudocontinuous arterial spin labeling (3D-pcASL) and suggested that the ability of 3D-pcASL perfusion imaging in distinguishing between pseudoprogression and tumor recurrence in GBM patients is almost the same as that of DSC, but 3D-pcASL is superior to DSC when the lesions are disturbed by magnetic susceptibility artifacts (44, 45). The reason is that the fast spin echo (FSE) technology used in GE 3D-ASL can effectively overcome the disadvantages of DSC being vulnerable to susceptibility artifacts. The artifacts can attenuate the imaging signal, usually when the focus is on the skull base, paranasal sinuses or large surgical resection cavity with blood residue. Another deficiency of DSC imaging is that the contrast medium may leak into the space where the BBB is destroyed. When it happens, the values of rCBV parameters cannot reflect the real perfusion level (46). Also, there is no unified standard between different imaging parameters and postprocessing methods. These factors will affect the diagnostic accuracy of DSC perfusion imaging to varying degrees.

**Figure 1:**
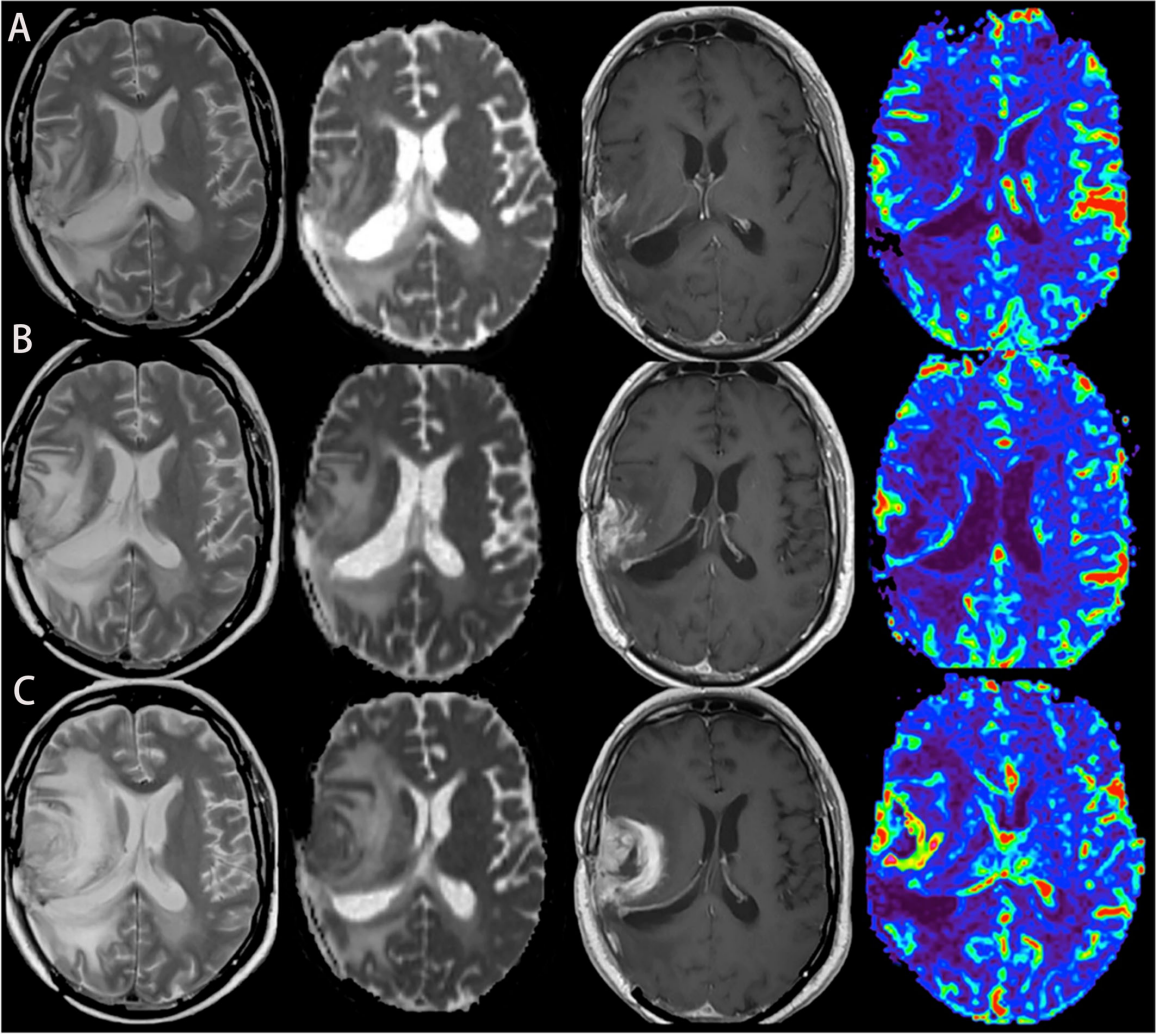
A case of glioblastoma relapsed during immunotherapy, T2, ADC map, T1-enhanced, and CBV map from left to right. **(A–C)** MRI was performed in the 2nd, 6th, and 8th months of immunotherapy, respectively, showing that the edema degree of the lesion was gradually aggravated, the enhancement was more obvious, and the perfusion was higher.

So far, there are still few researches about the application of PWI on assessing the immunotherapeutic response of GBM. In a study of advanced MRI assessing dendritic cell immunotherapy against GBM, it was found that the difference of relative cerebral blood volume (△rCBVmax) could effectively differentiate tumor recurrence from pseudoprogression, with a sensitivity of 67% and specificity of 75% (*p* = 0.004), suggesting that the value of △rCBV might be more helpful to distinguish them than the absolute value of rCBV during follow-up (29) (**Figure 1**). Research by Vrabec et al. showed that the maximal rCBV ratios in the contrast-enhancing area were potential radiological indicators to distinguish between inflammatory response induced by immunotherapy and tumor recurrence (30). Another follow-up study on immunogene-treated glioblastoma multiforme with DSC perfusion imaging combined with contrast-enhanced MR imaging also supported this view (31).

It is worth noting that both DCE-MRI and ASL techniques have not been widely explored in GBM patients treated with immunotherapy, which may be due to the lack of standardized acquisition parameters of DCE-MRI and the poor image signal of ASL perfusion imaging. However, these two perfusion techniques still have their own advantages. For instance, DCE-MRI can measure vascular permeability by pharmacokinetic parameters to quantify the movement of contrast media through BBB (47, 48). Compared with DSC-MRI, the ability of DCE-MRI of quantifying the permeability can make the calculation of cerebral blood volume more precise. 3D-pcASL can avoid the influence of magnetic susceptibility artifacts. If we could combine the advantages of various perfusion imaging to make up for the shortcomings, we would have a powerful supplementary tool to evaluate immunotherapeutic response in GBM.

### Diffusion Imaging

**Figure 2:**
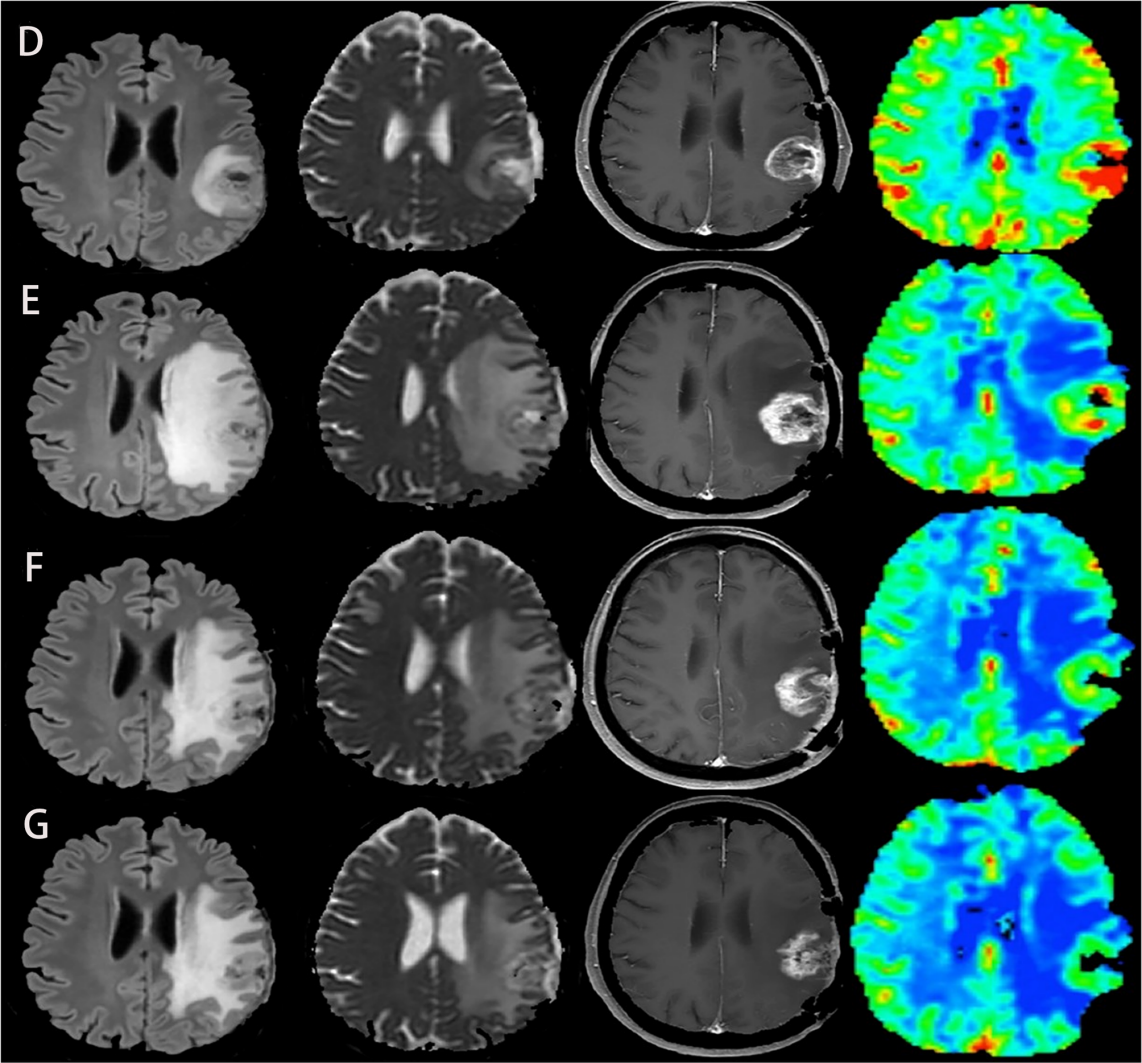
Another case of glioblastoma developed pseudoprogression during immunotherapy, with FLAIR, ADC map, T1-enhanced, and CBV map from left to right. **(D–G)** MRI was performed before immunotherapy and 2, 4, and 6 months after immunotherapy, respectively. Although tumor recurrence was suspected at the second month, the subsequent two MRI showed that the lesion became smaller, the degree of edema and imitation of diffusion alleviated, and the perfusion decreased. These two cases demonstrate that the combination of conventional MRI and advanced MRI imaging can accurately identify pseudoprogression and tumor recurrence of glioblastoma after immunotherapy. The above two figures were reproduced with the permission of (29) (Copyright at Multidisciplinary Digital Publishing Institute).

Diffusion-Weighted Imaging (DWI) reflects the diffusion of water molecules in the tissue of interest. The most widely used quantitative parameter is the apparent diffusion coefficient (ADC), which is inversely proportional to the cell density (49, 50). Based on this characteristic, it has been used in tumor identification, grading, and therapeutic response monitoring (51–54). In patients with recurrent gliomas, the diffusion of water molecules within the tumor was limited and the ADC values decreased, while treatment-related response, such as pseudoprogression, had higher ADC values than recurrent gliomas. This point of view was confirmed by a meta-analysis of diffusion magnetic resonance imaging combined with ADC measurements for distinguishing between glioma recurrence and pseudoprogression. Six cohort studies were included in the meta-analysis, and different ADC values were analyzed, including mean ADC values, relative ADC (rADC), and 5th percentile values. The results proved that the ADC values of pseudoprogression was higher than that of tumor recurrence, which provided a reliable foundation for the differentiation of the two (55). To date, some researches have applied this technique to the assessment of glioma immunotherapy and studied the evaluation effect of different ADC values. Song et al. conducted a retrospective study of 19 patients with recurrent GBM to evaluate whether the early changes in the quantitative parameters of diffusion and perfusion MRI before and after immunotherapy can determine the treatment-related changes. They calculated the rADC values and several perfusion parameters of the lesions before and after treatment and found that only the change of rADC could be used as an early marker to evaluate the response within 6 months after treatment (33). Another study also proved that rADC could help predict the immuno-therapeutic response and survival rate in patients with GBM (29) (**Figure 2**). Moreover, serial parametric response mapping of ADC performed at multiple time points of therapy may help identify pseudoprogression as an imaging biomarker in vaccine therapy for pediatric diffuse intrinsic pontine glioma (34). However, some studies believe that the application of the mean ADC values on differentiating pseudoprogression from tumor recurrence has some limitations, because the ADC values of cystic and necrotic areas are higher than that of solid tumors, which will affect the accuracy of the final results. It is considered that the 5th percentile values are better for the distinction (56, 57). Although ADC has good diagnostic value as a whole, the practicability of these different ADC parameters needs to be further studied. In addition, these results need to be verified in multicenter and larger cohorts.

Another kind of imaging technique commonly used in clinic is diffusion tensor imaging (DTI), which uses the diffusion anisotropy of water molecules for imaging. The fractional anisotropy (FA) images can show the structure and anisotropy of white matter fibers in the brain, and the change of FA can evaluate the therapeutic effect. Wang et al. combined DTI and DSC-MRI and found that the best models to distinguish between true progression and none-true progression (pseudoprogression and mixed progression) included FA, linear anisotropy coefficient (CL), and rCBVmax. It is suggested that the combination of DTI and DSC perfusion parameters could help evaluate the therapeutic response of gliomas (58). Although DTI has not been applied to the evaluation of immunotherapy in GBM, a recent study on the association of T cell density and diffusion tensor MRI changes in brain metastases revealed that FA in the peritumoral region was closely related to the density of CD^3+^ T-cell infiltration, indicating that FA could reflect the tumor immune microenvironment. This finding supports future researches and can be used to detect the sensitivity of neurological tumors to immunotherapy (59).

Furthermore, researchers also explored the role of some advanced diffusion models in assessing therapeutic response of brain tumors. These techniques are mainly used in scientific researches, such as intravoxel incoherent motion (IVIM) MRI, restriction spectrum imaging (RSI) MRI, etc. Based on the double exponential model, IVIM can simultaneously obtain diffusion and perfusion parameters reflecting tumor cellularity and vascularity. Fast diffusion coefficient (D*) mainly reflects perfusion information, slow diffusion coefficient (D) represents real diffusion information, and perfusion fraction (f) reflects blood flow (60). At present, it has been successfully used in gliomas for grading and distinguishing between treatment-related changes and tumor progression (61–63). However, this technique has not been used to monitor the response to immunotherapy yet. RSI-MRI is an advanced DWI technique, which provides a direct method for measuring tumor cellularity *in vivo* (64). Compared with the traditional DWI model, RSI can improve the conspicuity and delineation of high-grade tumors (65), better distinguish between true and pseudoresponse in antiangiogenic therapy (66), and better display white matter tracts in peritumoral edema areas (67). These advantages indicate that RSI-MRI have a good application prospect in the immunotherapy of neurological tumors. In a case report of immunotherapy for GBM, authors demonstrated that RSI could differentiate between pseudoprogression and tumor relapse while conventional DWI imaging could not provide more information (36). Despite that these advanced DWI techniques can provide better tissue structural characteristics than traditional DWI, the potential pathophysiological mechanism of tumor is still unknown.

### Amide Proton Transfer

Currently, APT is a relatively popular MR molecular imaging technique that can quantify free proteins with noninvasion and nonradiation. It reflects the changes of concentration and environment by detecting amide proton (NH) in endogenous low-concentration proteins or peptides. This technique displays its application value in a variety of central nervous system diseases (68–73) and shows great potential in glioma grading and curative effect evaluation (74, 75). Ma et al. used three-dimensional APT imaging technique combined with several conventional MRI sequences to evaluate the imaging features of tumor recurrence and pseudoprogression in 32 patients with gliomas who received standard treatment. It was found that the two kinds of progression had similar performance on conventional MRI. On the contrary, patients with tumor recurrence exhibited high signal intensity (relative to contralateral normal brain tissue) on APT-weighted (APTw) images, while patients with pseudoprogression showed equal to mild hyperintensity on APT-weighted images. Quantitative results demonstrated that compared with conventional MRI sequences, APTw could greatly improve the ability of MRI to distinguish between pseudoprogression and tumor recurrence (76). Additionally, as the therapeutic benefit and prognosis of glioma are related to its molecular subtypes and the expression of some proteins, APT imaging can detect the expression of MGMT protein before operation and provide relevant information for the possible drug resistance during treatment and the corresponding targeted therapy (77).

APT also has some shortcomings. In APTw images, red represents higher protein content, but not all red areas represent lesions or high-grade gliomas. Some tissues present high signal intensity on APTw images as well, like fat, cysts, and blood vessels. In addition to gliomas, there are other lesions that may also show high signal intensity, such as meningiomas, lymphomas, and some metastases. Most APTw images remove skull information because of the high signal of skull, which may hide potential lesions near the cerebral cortex. For this reason, using APT imaging alone to judge the nature of lesions may not be accurate enough, and it is best to combine multiple sequences to make a comprehensive diagnosis. Up to now, no research has reported the use of APT in the evaluation of immunotherapeutic response in GBM, but previous studies have shown that APT imaging is of great help to improve the diagnostic accuracy. If it is to become a powerful tool to assess immunotherapeutic response, it is necessary to continue exploiting and developing this technique and carrying out more clinical and scientific researches.

### Magnetic Resonance Spectroscopy

MRS uses the phenomenon of magnetic resonance chemical shift to determine the molecular composition of substances. It can simultaneously measure the concentrations of several metabolites in brain tissue and tumors and can be used to diagnose, grade, and evaluate the curative effect of brain tumors (78). The metabolism of brain tumor is exuberant, while that of chronic inflammation is lower. From the metabolism degree of lesion, we can decide its composition and distinguish between benign and malignant tissues (79, 80). The typical proton magnetic resonance spectroscopy (1H-MRS) manifestation of glioma exhibits obvious inversion of Cho/NAA ratio, while inflammatory lesions are characterized by increased Cho/Cr ratio and normal or decreased NAA/Cr ratio (81, 82). Thus, the response induced by immunotherapy and tumor progression in glioma patients can be distinguished by the concentration of metabolites. Floeth et al. found that the metabolic data of MRS may help to distinguish between tumor recurrence and pseudoprogression after local immunotherapy of GBM and contribute to further decision-making (37). In addition, a recent meta-analysis suggested that among the advanced MRI techniques, MRS had the highest diagnostic accuracy in distinguishing between treatment-related changes and tumor recurrence, with a sensitivity and specificity of 91% and 95%, respectively, which showed the good diagnostic performance of MRS (43).

MRS has some limitations in detecting small lesions compared with other MR imaging techniques due to its low spatial resolution, and it needs to be collected in high quantities because of the low concentration of metabolites in tumor tissues, which needs more time. The determination of metabolite concentration may also be affected by MR equipment, pulse sequence and data postprocessing methods. Lastly, MRS requires experienced operators to define exactly areas of interest, which is faced with technical challenges in clinical practice (78).

### Positron Emission Tomography

Positron emission tomography-computed tomography (PET-CT) is a metabolic functional imaging technique, which is applied to diagnose and analyze lesions by imaging radioactive markers. It is commonly used in clinical tumor staging, curative effect evaluation, and therapy. The most widely used PET tracer is 18F-fluorodeoxyglucose (18F-FDG) based on glycolysis, whose tracer concentration occurs in hypermetabolic lesions. Every technology has some deficiencies, and FDG-PET is no exception. First of all, the resolution of PET is relatively low, and normal brain tissue also shows high metabolism. If the lesion is close to the cerebral cortex, the measured FDG uptake value cannot reflect the true condition of the lesion. Furthermore, treatment-related necrotic reactions can also be characterized by increased glucose metabolism, resulting in increased FDG uptake (83). Although FDG-PET is widely used in clinic, it may be for some reasons above that make the accuracy of differential diagnosis of tumor recurrence and pseudoprogression not high (84, 85). Therefore, radioactive tracers with higher tumor-background uptake ratio have been studied.

Due to the increased proliferative activity and amino acid transport of malignant brain tumors, and the relatively low level of amino acid uptake in normal brain tissue, the use of amino acid-based radioactive tracers can improve the tumor-background ratio to some extent and identify tumors better (86). Till now, some radioactive tracers based on amino acids have been developed, such as ^11^C-methyl-l-methionine (^11^C-MET) and O-(2-[18F]fluoroethyl)-l-tyrosine (^18^F-FET). Studies have indicated that these two tracers have good accuracy in making a distinction between treatment-related response and tumor recurrence, and their manifestations are similar (87). However, ^11^C-MET is difficult to be commonly used in clinic owing to its short half-life and difficulties of preparation. Contrarily, 18F-FET has a long half-life, and the preparation process is relatively easy. In a study of immunotherapy with DC vaccination in GBM patients, 18F-FET PET imaging showed a more accurate identification ability than that of contrast-enhanced MRI initially (38). Although this study had several limitations such as a small sample size, it pointed out that 18F-FET PET had a potential role in monitoring the immunotherapy efficacy of GBM. In addition, Joseph et al. speculated that the PET probe for deoxycytidine kinase (dCK) could be used to distinguish between immune inflammatory response and enhancement foci caused by other factors in contrast-enhanced MRI imaging. They applied DC vaccination and/or PD-1 mAb blockade therapy to mice with orthotopic malignant gliomas model and GBM patients, and then used dCK PET probe and contrast-enhanced MRI for imaging respectively. The ratio of MRI contrast enhancement region to PET probe uptake area (immunotherapeutic response index) was used to describe the immune inflammatory activity in tumors. Finally, it was found that the accumulation of dCK PET probe in tumors and secondary lymphoid organs increased after immunotherapy, indicating that the immunotherapeutic response could be quantified by combining dCK PET probe with MRI imaging, which could be a potential biomarker for monitoring tumor immunotherapy (39).

With the gradual development of PET/MRI, the combination of PET and MRI makes full use of the good soft tissue contrast and multi-parameter evaluation ability of MRI. Compared with PET/CT, PET/MRI has superiority in the diagnosis and characterization of several diseases (88). Researchers have found the potential of PET/MRI in evaluating therapeutic response of GBM (89, 90) and the potential benefit of F-18 fluorothymidine (FLT)–PET/MRI for the diagnosis of melanoma brain metastasis and treatment monitoring of targeted therapy and immunotherapy (91). The ability of PET/MRI imaging in monitoring the treatment response to immunotherapy of GBM needs to be further studied. The future of PET/MRI is bright, and any new techniques need lots of researches to prove its value in clinical application.

## Application of Artificial Intelligence in Immunotherapy of Glioma

AI has developed rapidly in medical field in the past decade, especially in image identification. Many studies have reported the application of AI in diagnosis, grading, curative effect evaluation, and overall survival prediction of glioma, showing the great superiority of AI technology (92–99). Among them, radiomics is a new field that uses automatic data mining algorithm to transform a large number of image data into high-dimensional feature space. In the identification of treatment response and tumor progression in glioma, studies have investigated that the diagnostic performance of multiparameter radiomics model is better than single parameter model. The former can find more hidden information in the image data of glioma and improve the treatment of patients (100). The expression status of genes related to the prognosis of GBM can also be predicted from the features extracted from radiomics (101). Furthermore, radiogenomics, which combines imaging features with genome maps, is also helpful to find prognosis-related immune biomarkers (102). Despite the rapid development of AI, there are still some problems. Any algorithm needs to provide a large amount of high-quality data, and many researches on AI have a small amount of data, poor quality, and lack unified standards. These are the problems that need to be solved in the future.

## Conclusion

Pseudoprogression is the main problem that needs to be tackled in the treatment process of GBM, and the identification of which is also essential for the follow-up treatment. However, current assessment of treatment response of immunotherapy is still in the exploratory stage and does not meet the standard of routine clinical use. Fortunately, establishing a standard imaging scheme is the key to reverse this situation. The advanced imaging techniques have been widely studied and used as a tool to evaluate the therapeutic response in GBM. A large number of studies supported that the combination of various advanced imaging techniques can improve the diagnostic accuracy, expanding our prospective to the development of multimodal imaging. As for now, however, these imaging methods need to be further verified in multicenter and large sample clinical trials to drive them to truly become powerful diagnostic tools in the future.

## Author Contributions

YL and YM provided the draft, evaluated the literature, and wrote the review. MW, BS, SL, and LC revised the review. RX, FZ, and HC contributed to the review. All authors contributed to the article and approved the submitted version.

## Funding

This work was supported by the Natural Science Foundation of China (Grant No. 81501462); the Chengdu International Science and Technology Cooperation Funding (Grant No. 2019-GH02-00074-HZ); the 1·3·5 Project for Disciplines of Excellence-Clinical Research Incubation Project, West China Hospital, Sichuan University; and the Functional and Molecular Imaging Key Laboratory of Sichuan Province (Grant No. 2012JO0011).

## Conflict of Interest

The authors declare that the research was conducted in the absence of any commercial or financial relationships that could be construed as a potential conflict of interest.

## Publisher’s Note

All claims expressed in this article are solely those of the authors and do not necessarily represent those of their affiliated organizations, or those of the publisher, the editors and the reviewers. Any product that may be evaluated in this article, or claim that may be made by its manufacturer, is not guaranteed or endorsed by the publisher.
